# A Review of Machine-Vision-Based Analysis of Wireless Capsule Endoscopy Video

**DOI:** 10.1155/2012/418037

**Published:** 2012-11-13

**Authors:** Yingju Chen, Jeongkyu Lee

**Affiliations:** Department of Computer Science and Engineering, School of Engineering, University of Bridgeport, Bridgeport, CT 06604, USA

## Abstract

Wireless capsule endoscopy (WCE) enables a physician to diagnose a patient's digestive system without surgical procedures. However, it takes 1-2 hours for a gastroenterologist to examine the video. To speed up the review process, a number of analysis techniques based on machine vision have been proposed by computer science researchers. In order to train a machine to understand the semantics of an image, the image contents need to be translated into numerical form first. The numerical form of the image is known as *image abstraction*. The process of selecting relevant image features is often determined by the modality of medical images and the nature of the diagnoses. For example, there are radiographic projection-based images (e.g., X-rays and PET scans), tomography-based images (e.g., MRT and CT scans), and photography-based images (e.g., endoscopy, dermatology, and microscopic histology). Each modality imposes unique image-dependent restrictions for automatic and medically meaningful image abstraction processes. In this paper, we review the current development of machine-vision-based analysis of WCE video, focusing on the research that identifies specific gastrointestinal (GI) pathology and methods of shot boundary detection.

## 1. Introduction

Wireless capsule endoscopy (WCE) is a technology breakthrough that allows the noninvasive visualization of the entire small intestine. It was made possible because of the recent advances in low-power and low-cost of miniaturized image sensors, application-specific integrated circuits, wireless transmission technology, and light emitted diodes. This swallowable capsule technology enables the investigation of the small intestine without pain or need for sedation, thus encouraging patients to undergo GI track examinations. The first WCE was launched by Given Imaging (PillCam SB; Yokneam, Israel) in 2001. The successful launch of WCE encourages several other capsule manufacturers to develop their own products.  Table 1 is a list of commercially available capsule specifications. According to Given Imaging, more than 1,200,000 patients worldwide have benefited from their PillCam endoscopy.

Although WCE allows access to the small intestine noninvasively, the average viewing time ranges between 1 and 2 hours, depending on the experience of the gastroenterologist. In order to assist the gastroenterologist to speed up the review session, machine vision researchers have proposed various systems, including automatic video summarization, general abnormality detection, specific pathology identification, shot boundary detection, and topographic video segmentation. In this paper, we review the current development of machine vision-based analysis of WCE video, focusing on the research of specific GI pathology detection and shot boundary detection. A review of current capsule endoscopy hardware development is available to the interested reader in [1]; the review of machine vision-based analysis for push enteroscopy, intraoperative enteroscopy, push-and-pull enteroscopy, and radiographic methods is beyond the scope of this paper.

## 2. Image Features for Abstraction

A color WCE image is a snapshot of the digestive tract at a given time. However, in a computer-aided diagnosis system, the image content semantics needs to be translated in numerical ways for interpretation. There are several ways to represent the numerical form of an image known as *image abstraction*. Among WCE applications, there are three popular features for image abstraction: (1) *color*, (2) *texture*, and (3) *shape* features. Color images produced by WCE contain much useful color information and hence can be used as effective cue to suggest the topographic location of the current image. 

Figure 1 shows typical images taken from each organ. In this figure, the stomach looks pinkish, the small intestine is yellowish due to the slightly straw-color of the bile, and the colon is often yellowish or greenish due to the contamination of the liquid form of faeces. Another popular image abstraction feature in medical-imaging-related applications is the texture feature [2]. In WCE applications, a unique texture pattern called “villi” can be used to distinguish the small intestine from other organs. In addition, abnormality in WCE video can be discriminated by comparing the texture patterns between normal and abnormal mucosa regions, making texture pattern a popular feature for image abstraction. Shape feature is another commonly used abstraction approach for machine vision applications. Object shapes provide strong clues to object identity, and humans can recognize objects solely on their shapes. In the following subsections, we provide a high level survey of these features along with some popular implementations. 

### 2.1. Color

Color is a way the human visual system used to measure a range of the electromagnetic spectrum, which is approximately between 300 and 830 nm. The human visual system only recognizes certain combinations of the visible spectrum and associates these spectra into color. Today, a number of color models (e.g., RGB, HSI/HSV, CIE Lab, YUV, CMYK, and Luv) are available. Among all, the most popular color models in WCE applications are the RGB and HSI/HSV color models.

The RGB color model is probably best known. Most image-capturing devices use the RGB model, and the color images are stored in forms of two-dimensional array of triplets made of *red*, *blue*, and *green*. There are a couple of characteristics that make the RGB model the basic color space: (1) existing methods to calibrate the image capturing devices and (2) multiple ways to transform the RGB model into a linear, perceptually uniform color model. On the other hand, the main disadvantage of RGB-based natural images is the high degree of correlation between their components, meaning that if the intensity changes, all three components will change accordingly [5].

The HSI/HSV color model is another commonly used model in machine vision applications. Three components, *hue*, *saturation*, and *intensity* (or *value*), can be obtained through simple transformations of the RGB model. Hue specifies the base color, saturation specifies the purity of a color, and intensity shows how bright the color should be. Although the HSI/HSV model carries the same shortcomings as the RGB model, Gevers et al. show that HSI model is invariant to viewing orientation, illumination direction, and illumination intensity [23]. This outstanding property has made HSI/HSV model much less sensitive to illumination changes, which is a common problem of the WCE image as the battery of the capsule weakens over time. 

### 2.2. Texture

Texture is a fundamental property of surfaces. It can be seen almost anywhere (e.g., tree bark, grass, sand, wall, etc.). Some examples of texture analysis applications are industrial surface inspection, biomedical image analysis, and face and facial expression recognition. A common approach for discriminating WCE images is to extract mucosa texture and then classify the feature with trained classifiers. Texture feature extraction in WCE imaging is difficult because: (1) WCE images are taken by a miniature camera which has a limited range of luminance and hence suffer from illumination variations; (2) as a tiny capsule travels down the GI tract via digestive peristalsis, the moving or spinning motion of the capsule contributes to uneven illumination; (3) the battery life weakens over time; and (4) the uncertainty of a functioning digestive tract such as food residue, bubbles, faeces, and so forth is encountered. Because of these challenges, the most popular textural features are the multi-resolution and gray scale texture features.

In general, the characteristics of texture are measured by variations in the image's intensity or color. The differences between the gray level value of a reference pixel and its neighboring pixels have been used for analyzing textural properties. *Local Binary Pattern* (LBP) operator, proposed by Ojala et al. in [24], is one of the texture features that are invariant against gray scale transformation and rotation, yet computationally simple. In order to compute the texture model of a specific surface, an LBP code is computed for each pixel of this surface by comparing its gray level against those of its neighboring pixels. The final histogram of LBP codes is the texture model that represents this surface.  Figure 2 is an example of a texture model that utilizes a joint LBP histogram to represent the mucosa of different organs.

Another well-known texture feature called *Gray Level Co-occurrence Matrices* (GLCM) was introduced by Haralick et al. in the 1970s [25, 26]. It belongs to the second-order statistics methods that describe spatial relationships between the reference and neighbor pixels within a local neighborhood. In this approach, texture is characterized by the spatial distribution of gray levels (or gray scale intensities) in a neighborhood. A cooccurrence matrix is defined to represent the distance and angular spatial relationship over subregion of a gray-scale image. It is calculated to show how often the pixel with gray level value occurs horizontally, vertically, or diagonally to adjacent pixels. Once the GLCMs are created, the similarity of texture pattern can be measured using the formulas as described in [25, 26].

As the size of lesion may vary in size, it is desirable to analyze the lesion and its mucosa in multiple resolutions. *Wavelet* theory has been commonly used in multiresolution analysis. In this method, an image is analyzed at various frequencies under various resolutions. Wavelet transform provides powerful insight to the spatial and frequency characteristics of an image. In image processing, the transform could be achieved using Discrete Wavelet Transform (DWT) by decomposing an image into four subbands: LL1, LH1, HL1, and HH1 (Figure 3(a)). The LL1 subband is referred to as the *approximation component* while the remaining subbands are referred to as the *detail components*. Subband LL1 could be further decomposed to result in a two-level wavelet decomposition (Figure 3(b)). By repeating this process with subband LL2, we can obtain the three-level wavelet decomposition. This process can be repeated on the approximation component until the desired scale is reached. The coefficients in the approximation and detail components are the essential features for DWT based texture analysis and discrimination.

### 2.3. Shape

In WCE imaging, shape-based features are employed mostly in polyp and tumor detection. In this category, the most common process is to detect the edges first, followed by region segmentation. Geometric rules are applied to the segmented regions to construct the shape information of these regions. Although most of the objects in the real world are three-dimensional, image and video processing usually deals with two-dimensional projections of real world objects. Good descriptors should capture shape characteristics in a concise manner, and they should be invariant to scaling, rotation, translation, and various types of distortions. In addition, they should be able to handle nonrigid deformations caused by perspective transformation of two-dimensional shapes. 

In machine vision, *moments* describe image content with respect to its axes. For example, the *width* of a set of points in one dimension or the *shape* of a cloud of points in a higher dimension can be measured by computing the second moment of these points. Since moments describe image content with respect to its axes, the global and detailed geometric information of an image can be captured by moments.

*Gabor* filters have been widely used for edge detection. Gabor filters are defined by harmonic functions modulated by a Gaussian distribution. Since *frequency* and *orientation* representations of Gabor filters are similar to those of the human visual system, a set of Gabor filters with different frequencies and orientations are found to be useful for texture-based region segmentation of an image [27]. In [28], Field introduces Log-Gabor filters and suggests that natural images are better coded by filters that have Gaussian transfer functions when viewed on the logarithmic frequency scale. Unlike Gabor filters that suffer from bandwidth limitation, Log-Gabor filters can be constructed with arbitrary bandwidth and hence can be optimized to produce a filter with minimal spatial extent. 

## 3. Computer-Aided Diagnosis Systems

**Figure 4:**
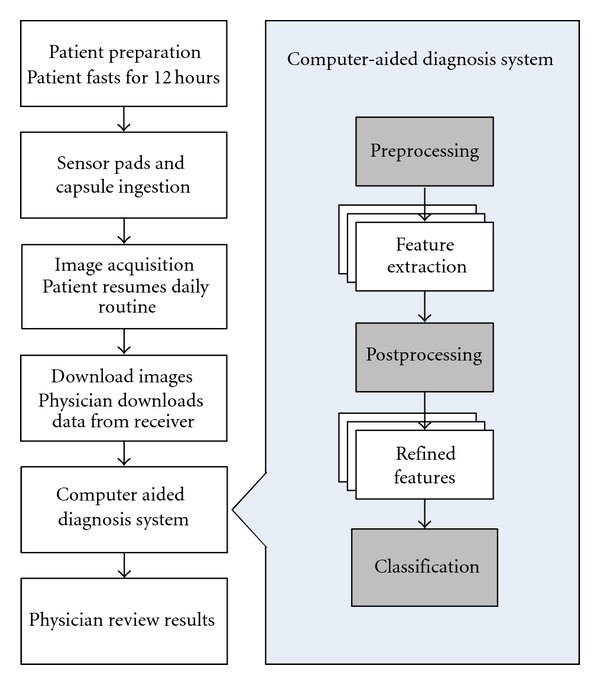
General steps involving in computer-aided diagnosis (CAD) system where gray boxes may be optional.

Figure 4 is an illustration of the general process flowchart of a typical WCE machine vision analysis system. It is a common practice that each image is preprocessed to enhance the accuracy of feature extraction, followed by feature refinement. The output of the feature refinement is a concise form of the image abstraction for the final classification, and the classifiers may be artificial intelligent based or rule based. In general, artificial-intelligent-based classifiers need to be trained before use, whereas rule-based classifiers utilize “*if… then…*” rules for classification which requires no training.

### 3.1. Shot Boundary Detection and Video Segmentation

Temporal video segmentation is usually the first step towards automatic annotation of image sequences. It divides a video stream into a set of meaningful segments called *shots* for indexing. In conventional video segmentation, there are two types of shot transitions: *abrupt* and *gradual*. Abrupt transition is easier to detect because it occurs when the camera is stopped and restarted [29]; however, the detection of gradual transition is more difficult because it is caused by camera operations such as zoom, tilt, and pan. Although there are algorithms for shot transition detection, these are not suitable for WCE. WCE video is created without any stops; therefore, these algorithms do not work well in WCE. Instead of modeling shot boundary detection by camera operations, it is preferred that shots are modeled by similar semantic content or by the digestive organ.

**Figure 5:**
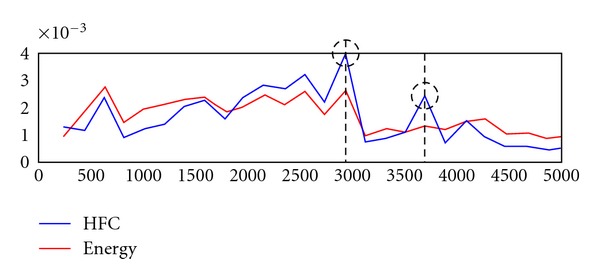
A shot detection scheme based on event boundary detection approach as described in [3].

In order to provide the gastroenterologist with a quick glance of the video contents, WCE researchers utilize digestive peristalses and image analysis techniques for shot boundary detection and organ boundary detection. Vu et al. proposed a coherent three-stage procedure to detect intestinal contractions in [30]. They utilized changes in intestinal edge structure of the intestinal folds for contraction assessment. The output is contraction-based shots. Another shot detection proposed by Iakovidis et al. was based on nonnegative matrix factorization (NMF) [31]. A full length of WCE video was uniformly sampled to generate consecutive nonoverlapping video segments followed by a dimensionality reduction algorithm. Fuzzy C-means was applied for extraction of most representative frames and the results were enhanced by applying symmetric NMF to the symmetric matrices. The final cluster indicators (or shots) were obtained using nonnegative Lagrangian relaxation. Another shot detection scheme based on organ was proposed by Mackiewicz et al. in [32]. The authors utilized three-dimension LBP operator, color histogram, and motion vector to classify every 10th image of the video. The final classification result was assessed using a 4-state hidden Markov model for topographical segmentation. In [3], two color vectors that were created with hue and saturation components of HSI model were used to represent the entire video. Spectrum analysis was applied to detect sudden changes in the peristalsis pattern. Chen et al. assumed that each organ has a different peristalsis pattern and hence, any change in the pattern may suggest an event in which a gastroenterologist may be interested (see Figure 5). Energy and High Frequency Content (HFC) functions are used to identify such change while two other specialized features aim to enhance the detections of duodenum and cecum. 

### 3.2. Significant Medical Event Detection

The primary use of capsule endoscopy is to find the source of bleeding and abnormality in the small intestine. Shot boundary detection may help to speed up the review by grouping images with similar semantics, but it may not detect medical significant events other than acute bleeding. A common trait of aforementioned algorithms is to group images by global features such as the distribution of color; however detail features such as patches of mucosa deficiency are often neglected. Most of medical significant events of WCE only account for a handful of images of the entire video, lesions in these images may not result in significant shifts in the global features and hence cannot be detected by shot boundary detection algorithms. Acute bleeding that lasts for several dozens of consecutive images may be bundled into one shot, since a vast amount of fresh blood may cause noticeable shifts among the global feature distribution. In order to detect significant medical events that contain little or no temporal relationship with their consecutive image frames (e.g., ulcers, polyps, tumors, etc.), imagewise semantic analysis is inevitable. In this subsection, we review the machine vision analysis literature on specific GI pathology identification, namely, *bleeding*, *ulcer*, *polyp*, and *tumor*.

The visual appearance of bleeding images is bright red. However, depending on the lifetime of blood, it could be black or tarry. Lau and Correia proposed a two-step system that discriminates bleeding and nonbleeding images under the HSV color model [6]. The classification is rule-based with certain combinations between color luminance and saturation. Their system classifies images into one of the following categories: (1) nonbleeding, (2) low-intensity bleeding, (3) bleeding, and (4) high-intensity bleeding. Another system proposed by Liu and Yuan [7] uses *Support Vector Machines* (SVMs) to classify images using color features extracted in the RGB color model. In [9], Penna et al. utilize a Reed-Xiaoli detector for bleeding regions and normal mucosa region discrimination. Karargyris and Bourbakis [10] propose a mechanism that combines Karhunen-Loeve color transformation, fuzzy region segmentation, and local-global graphs. Li and Meng [11] adopt neural network classifiers for bleeding imaging classification. The feature vector consists of color texture feature and statistical measures. The color texture feature is obtained from chrominance moment, while the statistical measures were obtained from the uniform LBP histogram. 

**Figure 6:**
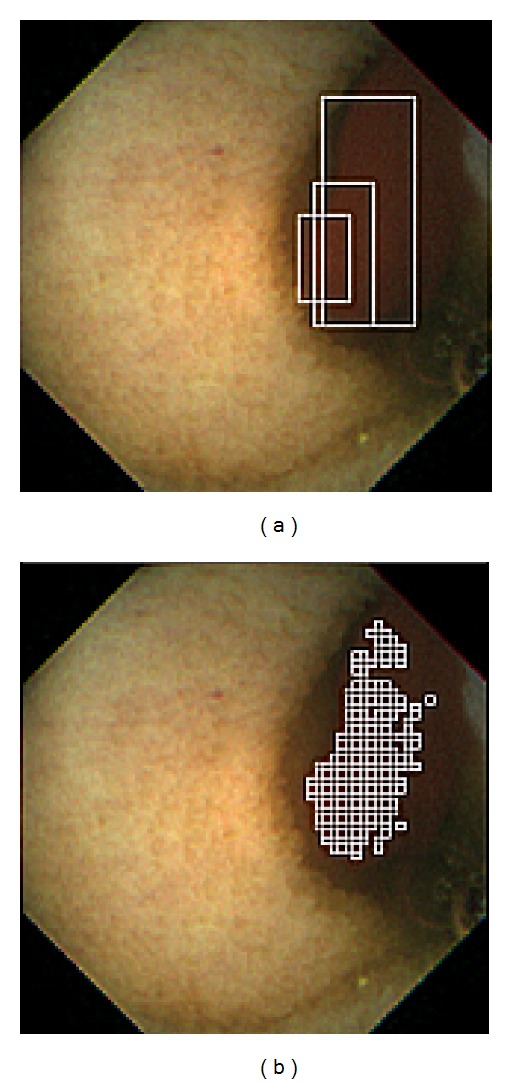
A bleeding detection scheme as described in [3].

The physician usually observes GI bleeding based on a set of medical criteria such as potential causes or sources of bleeding, bleeding frequency, and amount of blood loss. To mimic this process, Chen et al. [3] use a macro-micro hybrid approach for bleeding detection (see Figure 6). Unlike a binary classification approach, their system shows a potential bleeding distribution. In the macroapproach, each potential bleeding area is extracted using pyramid segmentation. Depending on the angle of the hue component, each suspicious bleeding segment is assigned with various weights for bleeding assessment. In the microapproach, each image is divided into 7 × 7 blocks and each block is validated against specific ranges of hue and saturation components for bleeding detection. Each image is analyzed using the two approaches and the final bleeding distribution is the average score of the two approaches. 

With regard to polyp detection, Karargyris et al. [17] suggest a rule-based classification system that utilizes log Gabor filters and SUSAN edge detectors. In [18], Li and Meng conduct a comparative study between two-shape features, MPEG-7 region based shape descriptor and Zernik Moments. The classifier is based on multilayer perceptron (MLP) neural networks. The authors conclude that Zernik Moments are superior to MPEG-7 region based shape descriptors.

With regard to tumor detection, Karkanis et al. [21] utilize a texture feature for tumor discrimination and the classifier is a multilayer feed forward neural networks (MFNNs). In [22], Li and Meng propose a feature vector consisting of LBP and DWT. The experiment result shows that their proposed feature outperforms both original rotation invariant LBP and color wavelet covariance texture features.

**Figure 7:**
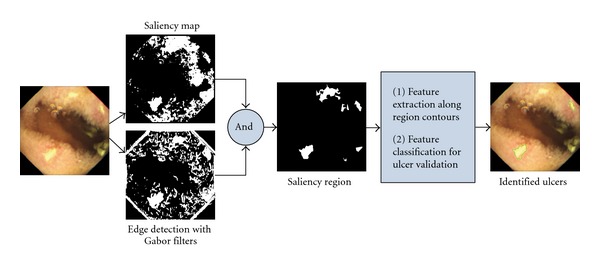
Ulcer detection scheme described in [4].

For ulcer detection, Li and Meng [13, 14] propose a feature vector that consists of curvelet transformation and uniform LBP. However, this approach performs an exhaustive comparison regardless of the unique visual appearance of an ulcer and hence, the performance could be slow. On the other hand, Karargyris and Bourbakis [15] present a segmentation scheme utilizing log Gabor filters, color texture features, and an SVM classifier. Although the authors considered the unique visual appearance of ulcers, the HSV color model they chose suffers the same shortcoming as the RGB color model. Chen and Lee [4] propose a four-step detection scheme (see Figure 7). The first step is to create a saliency map emphasizing the ulcerated mucosa, followed by initial saliency segmentation. Second, Gabor filters are applied to find the contour for saliency region refinement. When the saliency region is refined and extracted, a feature vector is formed using LBP and six statistical measurements along the region contour. Finally, the feature vector is validated by an SVM classifier for ulcer detection. 

## 4. Discussion

Human beings are capable of interpreting images at different levels. For example, we can interpret images based on low level features such as color, shape, texture, or based on high level semantics such as events or abstract objects. A computer only interprets images based on low level features and thus, choosing the right image feature becomes critical for computer-aided diagnosis systems. Generally, feature selection for image abstraction is modeled to mimic a human's understanding of visual content. For example, the dominant color of fresh blood is red, hence it is desirable to use the color feature for bleeding detection. The color and amount of blood are important cues for bleeding detection; however, color alone does not always discriminate images correctly. According to experiment results from [3, 6–9, 11, 12], the accuracy of bleeding detection ranges from the lower 70% to the upper 90%. Bleeding image detection based on predefined threshold values is especially difficult since the image quality is susceptible to illumination and camera motion and also due to the fact that visual color appearance of an organ varies from person to person. 

Currently, detection rate for ulcer, polyp, and tumor is also not perfect. According to [12–15], the ulcer detection accuracy for image-based experiments ranges from 90% to 96%. The detection rate for polyp, as published in [16–18], ranges from 73% to 89%. As for tumor detection, the detection rate ranges from 96% to 98% [19–22]. Today, the image quality of WCE imaging is considered low when compared to conventional endoscopes. Commercially available WCEs have very limited flexibility in terms of capsule control. The hardware design limitation of WCE (e.g., battery life, no manual operation support, etc.) and uncertainty operating inside a functioning digestive tract (e.g., food residue, faeces, contraction, etc.) make developing an efficient computer aided diagnosis system a challenging task. Although innovative approaches are introduced to enhance the quality of WCE images, the accuracy of detection remains low when compared to other image processing applications such as motion detection in surveillance video or industrial surface inspection. 

Meanwhile, the experimental results show a wide variance in accuracy, one possible cause could be the size of image collection. Although a full length WCE video contains at least 55,000 images, most of the experimental results that claim high accuracy were tested with less than 1,000 images (Table 2). Among the literature in this paper, only [3, 8, 32] used full-length videos, other works were validated against privately selected image set. The aforementioned hardware limitation of WCE coupled with the lack of publicly accessible WCE video database makes it difficult for researchers to measure their work against some baseline. In addition, the fact that only a handful of significant images observed in each video also makes it difficult to effectively characterize the visual appearance for pathology assessment.

The research of effective image retrieval from an image or video database has been active since 1970s. However, a robust image understanding at the machine level remains an open issue. In the 1970s, the most popular method was text-based search. In this approach, only images that are annotated with text are retrieved. If there were any error in the annotation, the result could be erroneous. In the 1990s, content-based image retrieval (CBIR) was introduced as an alternative to text search. Low level features such as color, texture, and shape features are extracted to search images with similar visual features. Although there are sophisticated algorithms to describe color, texture, and shape features, these low level features cannot be compared to the human cognitive concept of visual content. The gap between human and computational ability to recognize visual content has been termed the *semantic gap* [33]. Semantic-based image retrieval was introduced in 2000s to create semantic content representation for images. This method allows users to perform a text query based on the semantic they have in mind for image retrieval. However, to be able to describe an image in semantic terms as identified by users remains an open issue in the image processing world. In particular, we are yet to see any work that offers semantic-based image retrieval tool for physicians to query WCE videos.

## 5. Conclusion

WCE technology is fairly new and is originally intended for the detection of obscure GI bleeding. However, GI specialists are still uncovering other potential uses of WCE for abnormal indications. Nevertheless, the need for automatic GI pathology identification is in strong demand. In this paper, we reviewed a variety of works that are related to shot boundary detection and GI abnormality detection. The main image abstraction approaches for WCE video can be classified into three image features: color, texture, and shape features. Depending on the visual characteristics of each pathology targeted, a suitable feature form is selected for image abstraction. Currently, most bleeding-related applications utilize color-based features, while other GI disorders utilize texture and shape features. Among the surveyed literature, we focus on the research that identifies specific GI pathology. This way we can learn the relationships between GI pathology and machine vision. Despite the effort that researchers put for abnormality detection, it is almost impossible to compare the performance of different implementations due to the lack of a publicly accessible WCE video database. Consequently, machine vision researchers are forced to test their implementation against relatively small image sets and thus slows down the development of commercially available tools for WCE video review sessions.

## Figures and Tables

**Figure 1:**
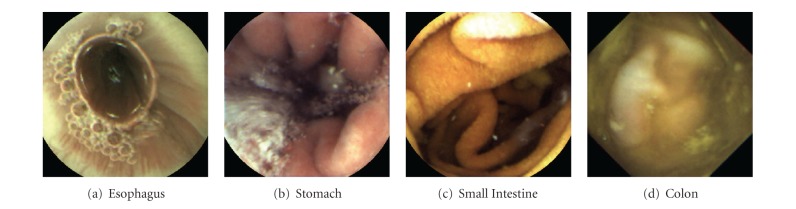
Typical images captured by WCE at different organs.

**Figure 2:**
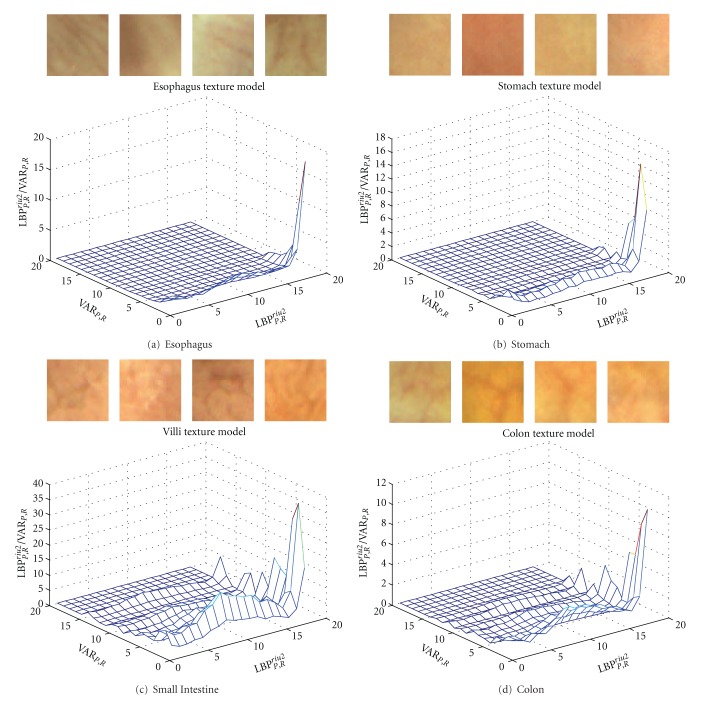
Mucosa representations based on a joint histogram of LBP operator (LBP_*P*,*R*_^*riu*2^) and local gray level variance (VAR_*P*,*R*_).

**Figure 3:**
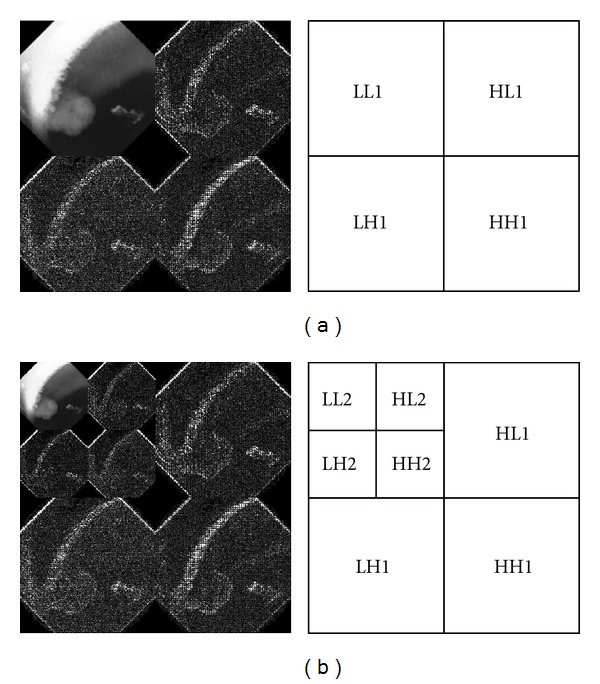
Image decomposition using DWT where (a) represents a one-level wavelet decomposition and (b) represents a two-level wavelet decomposition.

**Table 1:** Technical specifications of commercially available small intestine capsules.

Company	Given imaging Inc.	Olympus	IntroMedic	Jinshan
Capsule	PillCam SB/SB2	EndoCapsule	MiroCam	OMOM
Size (diameter × length)	11 mm × 26 mm	11 mm × 26 mm	11 mm × 24 mm	13 mm × 27.9 mm
Image Sensor	CMOS	CCD	CMOS	CCD
Resolution	256 × 256	NA	320×320	640 × 480
Field of View	140°/156°	145°	150˚	140 ± 10°
Image Capture Rate	2 fps	2 fps	3 fps	0.5–2 fps
Illumination	6 LEDs	6 LEDs	6 LEDs	6 LEDs
Battery Life	8 hr	8+ hr	11+ hr	8 ± 1 hr
Communication	RF	RF	HBC	RF
Approval	FDA 2001/2011	FDA 2007	FDA 2012	PRC FDA 2004

CMOS: complementary metal oxide semiconductor, CCD: charge-coupled device, LED: light-emitting diode, RF: radio frequency, HBC: human body communications, NA: not available.

**Table 2:** Summary of abnormality types, features and claimed experimental results.

Paper	Pathology				Feature			Experiment results		
	Bleeding	Polyp	Tumor	Ulcer	Color	Texture	Shape	Image count	Sensitivity	Specificity
Chen et al. [3]	*√*				*√*			1,857,657	71.54%	N/A
Lau and Correia [6]	*√*				*√*			1,705	88.30%	N/A
Liu and Yuan [7]	*√*				*√*			800	99.73%	98.89%
Giritharan et al. [8]	*√*				*√*			275,000	83.1%	93.6%
Penna et al. [9]	*√*				*√*			1,111	92%	88%
Karargyris and Bourbakis [10]	*√*						*√*	N/A	N/A	N/A
Li and Meng [11, 12]	*√*			*√*		*√*		200	92.6%	91%
Li and Meng [13, 14]				*√*		*√*		100	93.28%	91.46%
Karargyris and Bourbakis [15]				*√*			*√*	50	100%	67.5%
Iakovidis et al. [16]		*√*				*√*		4,000	87.5%	N/A
Karargyris and Bourbakis [17]		*√*					*√*	50	75%	73.3%
Li et al. [18]		*√*					*√*	300	89.8%	82.5%
Barbosa et al. [19]			*√*			*√*		192	98.7%	96.6%
Barbosa et al. [20]			*√*			*√*		600	97.4%	97.5%
Karkanis et al. [21]			*√*			*√*		N/A	N/A	N/A
Li and Meng [22]			*√*			*√*		300	97.33%	96%

Sensitivity = Number of True Postives/(Number of True Postives + Number of False Negatives). Sensitivity of 100% means no positives are incorrectly marked as negative. In other words, the test recognizes all positives.

Specificity = Number of True Negatives/(Number of True Negatives + Number of False Postives). Specificity of 100% means no negatives are incorrectly marked as positive. In other words, the test recognizes all negatives.

N/A: data is not available.
